# A Cross-Sectional Study of Individuals Seeking Information on Transient Ischemic Attack and Stroke Symptoms Online: A Target for Intervention?

**DOI:** 10.1371/journal.pone.0047997

**Published:** 2012-10-31

**Authors:** Anthony S. Kim, Sharon N. Poisson, J. Donald Easton, S. Claiborne Johnston

**Affiliations:** 1 Department of Neurology, University of California San Francisco, San Francisco, California, United States of America; 2 Department of Epidemiology and Biostatistics, University of California San Francisco, San Francisco, California, United States of America; University of Münster, Germany

## Abstract

**Background:**

Individuals with TIA/stroke symptoms often do not seek urgent medical attention. We assessed the feasibility of identifying individuals searching for information on TIA/stroke symptoms online as a target for future interventions to encourage urgent evaluation and we evaluated the performance of a self-reported risk score to identify subjects with true TIA or stroke.

**Methodology/Principal Findings:**

We placed online advertisements to target English-speaking adults in the United States searching for TIA/stroke-related keywords. After completing an online questionnaire, participants were telephoned by a vascular neurologist to assess the likelihood of TIA/stroke. We used logistic regression and the c-statistic to assess associations and model discrimination respectively. Over 122 days, 251 (1%) of 25,292 website visitors completed the online questionnaire and 175 were reached by telephone (mean age 58.5 years; 63% women) for follow-up. Of these participants, 37 (21%) had symptoms within 24 hours, 60 (34%) had not had a medical evaluation yet, and 68 (39%) had TIA/stroke. Applying a modified ABCD^2^ score yielded a c-statistic of 0.66, but 2 of 12 with a zero score had a TIA/stroke. Those with new symptoms were more likely to have TIA/stroke (OR 4.90, 95% CI 2.56−9.09).

**Conclusions/Significance:**

Individuals with TIA/stroke that are seeking real-time information on symptoms online can be readily identified, in some cases before they have sought formal medical evaluation. Although a simple self-reported risk score was unable to identify a low-risk population in this selected group, this population may still present an attractive target for future interventions designed to encourage urgent medical evaluation.

## Introduction

Transient ischemic attack (TIA) is associated with a high risk of subsequent stroke, particularly in the first hours after symptom onset. [1], [2] Although progress has been made to develop simple clinical scores to rapidly identify TIA patients at high risk of stroke [3] and to apply urgent interventions to prevent stroke after TIA, [4], [5], [6] most individuals with TIA do not seek urgent medical evaluation. [7], [8].

Knowledge of TIA among the general public is poor. [9] TIA or “mini-stroke”, as it is commonly known in the lay public, may not be recognized as a harbinger of stroke, particularly when symptoms are transitory or evanescent. [9], [10] So even when TIA or stroke is considered as a possibility, the importance of an urgent evaluation may not be fully appreciated. Furthermore, individuals seeking appropriate evaluation may be deterred by barriers to access to healthcare providers, particularly on weekends or evenings [11] or by the prospect of lengthy or costly emergency department visits for symptoms that may be perceived of as minor or transient. [12], [13] Therefore, improving early recognition of TIA and an appreciation for the substantial risk of stroke associated with TIA may help to reduce delays in presentation for medical evaluation and create additional opportunities to prevent stroke.

Within this context, the internet has become an increasingly important source of first-line medical information [14] in part due to widespread availability and relatively low barriers to access. [15] However, despite potential advantages in terms of cost, efficiency, and reach, internet-based public health interventions to target online information-seeking behavior have been underdeveloped and understudied.

Our ultimate goal is to reduce the burden of disease from stroke by developing and refining efficient interventions to empower individuals seeking information on TIA/stroke online to seek urgent medical attention. For the present study, our primary goal was to assess the feasibility of identifying a population of individuals that are searching for information on TIA/stroke online as a potential target for future interventions to encourage appropriate urgent medical evaluation. Our main target population was subjects with TIA since these patients are at higher short-term risk of developing a stroke than patients with prevalent stroke, but since the distinction between TIA and stroke is difficult to make by both clinicians and the lay-public, we also included patients with prevalent stroke. Our secondary goal was to assess the performance of a web-based self-assessment tool based on the ABCD^2^ score to identify TIA/stroke [16] in this population with the hope that this personalized information would be a more effective motivation for participants to seek appropriate medical attention than general advice on TIA.

## Methods

### Study Design

We conducted a cross-sectional study with recruitment, eligibility screening, enrollment, consent, and data collection activities accomplished online and with outcome assessments completed by telephone.

### Instrument Development and Pre-Testing

Candidate items for the online instrument were drawn from the Questionnaire for Verifying Stroke-Free Status; [17] components of the ABCD^2^ score (Age > = 60 = 1; Clinical Features: Speech Disturbance without weakness = 1, Unilateral Weakness = 2; Duration: 10–59 minutes = 1, >60 minutes = 2; Diabetes = 1); [1] and other items (frequency and timing of symptoms, suddenness of symptom onset, hypertension, and migraine history). The blood pressure item was omitted because this information would usually not be available by self-report for most participants. We pre-tested candidate items in 10 inpatients and 10 outpatients with confirmed TIA or minor stroke using semi-structured cognitive interviews to assess for comprehension and phrasing. This feedback was incorporated into the final instrument (see Text S1).

### Recruitment, Screening, Consent, and Enrollment

We placed a series of advertisements using an online marketing tool (Google Adwords, Google, Mountain View, California) to target users in the United States that were searching for keywords related to “mini-stroke”, “TIA”, “transient ischemic attack” or that were visiting webpages with related content that were part of an online advertising network (Google Display Network; See Text S2 for a list of targeted keywords). Users that clicked on an advertisement were taken to the study homepage (http://tia.ucsf.edu/), which provided information about the research study and basic information about stroke and TIA. Users that were interested in participating in the study then completed a seven-item screening questionnaire to confirm eligibility (symptoms concerning for a transient ischemic attack or “mini-stroke” within the last 6 months; age > = 18; ability to read, speak, and understand English; residence in the United States; and a willingness to complete the study questionnaire and the follow-up telephone assessment.) Those who met all entry criteria were asked to provide informed consent online. Consent was confirmed using a five-item post-test of comprehension. Those who were unwilling or unable to provide consent were excluded.

### Study Procedures

Participants provided contact information for the subsequent telephone assessment and then completed a 26-item questionnaire on demographic characteristics, medical history, and symptoms (See Text S3). We then scheduled a 15 minute telephone call with each participant in order to assess how likely the symptoms were due to TIA/stroke. These assessments were conducted by one of five vascular neurologists. Each neurologist was masked to the previously submitted responses to the questionnaire. Neurologists were free to elicit any information in order to assess the likelihood of TIA/stroke including self-reported neuroimaging findings. The likelihood of TIA/stroke was rated as “definitely not”, “unlikely”, “probable”, and “definite”.

TIA was defined as an acute neurological deficit lasting less than 24 hours attributable to focal brain ischemia, without evidence of a non-ischemic etiology and stroke was defined as a new neurologic deficit lasting over 24 hours with no apparent clinical or radiologic indication of a nonvascular mimic. To assess interrater reliability, half of the participants had a second independent telephone evaluation by a separate vascular neurologist that was masked to the results of the first assessment.

### Statistical Analysis

Based on questionnaire responses, we calculated a modified ABCD^2^ score for each participant during the analysis phase of the study. We used logistic regression to assess univariate and multivariable associations. Cronbach’s alpha was used to assess reliability across items assessing the same underlying construct (i.e. diabetes, hypertension, suddenness of symptom onset). We used a quadratic-weighted kappa to evaluate interrater agreement between the two neurologists.

### Ethics Statement

The University of California San Francisco Committee on Human Research approved this study. Consent was obtained for all participants online and the Committee specifically approved this form of consent as documented in an electronic database.

## Results

### Recruitment and Enrollment

A total of 4,600,427 advertisements were displayed over the 122-day enrollment period which resulted in 26,602 visits (average 218 visits/day) from 25,292 unique users. Total advertising costs for the study were $8,798.74–which corresponds to $0.35 per unique visitor or $35.05 per enrollee. All fifty states were represented as assessed by an Internet Protocol address-based geolocalization algorithm.

Fully 86% percent of visitors immediately exited the website within a few seconds of arriving at the home page, which is consistent with the notion that they clearly did not qualify for the study or were not interested in enrolling. Of the 291 visitors that initiated the enrollment process, 251 (86%; 1% overall) completed the eligibility screening, online consent, and questionnaire, which is not an unexpected yield rate for internet advertisements generally since there is trivial effort and little commitment for users to click on a link to find out more information. Of these, 175 (70%) were reached by telephone for follow-up. Reasons for failing to contact participants by telephone included the following: 31 did not respond to multiple telephone calls; 14 were initially reached by telephone to confirm their participation, but did not respond to subsequent telephone calls; 6 withdrew consent when reached; and 25 listed a nonworking or incorrect telephone number.

### Baseline Characteristics

The average age of participants was 59 and a majority were women (Table 1). A total of 52 participants (30%) were age 65 or older. Based on telephone area codes, participants from 40 states and the District of Columbia were represented. Vascular risk factors such as hypertension, diabetes, and a prior history of cerebrovascular disease were common. A prior history of migraine was also frequently reported.

**Table 1:** Characteristics of 175 users seeking information on Transient Ischemic Attack or Mini-Stroke Online.

	n = 175	
Age, mean (SD)	58.5	(12.5)
Female, n (%)	110	(63.2)
Diabetes, n (%)	33	(18.9)
Hypertension, n (%)	103	(58.9)
Prior TIA, n (%)	46	(26.3)
Prior stroke, n (%)	30	(17.1)
History of migraine, n (%)	64	(36.6)
Symptom onset, n (cumulative %)*		
within 24 hours	37	(21.8)
within 48 hours	54	(31.8)
within 1 week	88	(51.8)
within 1 month	131	(77.1)
within 6 months	170	(100.0)
Duration of symptoms, n (%)		
<10 minutes	54	(30.9)
10–59 minutes	46	(26.3)
60 or more minutes	71	(40.6)
Number of episodes within the past year, n (%)†		
1	71	(40.6)
2	29	(16.6)
3 to 5	24	(13.7)
6 to 10	18	(10.3)
11 or more	26	(14.9)
No previous episodes within the past year	104	(59.4)
Sudden onset, n (%)	102	(58.3)
Warning before episode, n (%)	33	(18.9)
Associated headache, n (%)	79	(45.1)
Speech symptoms, n (%)	95	(54.3)
Weakness, n (%)	63	(36.0)
Sensory symptoms n (%)	90	(51.4)
Double vision, n (%)	46	(26.3)
Had Not Sought Medical Attention	71	(40.6)
ABCD^2^ score, mean (SD)‡	2.7	(1.4)

* n = 170; † n = 168; ‡ ABCD^2^ score: age > = 60 = 1 point; Clinical Features: Speech Disturbance without weakness = 1 point, Unilateral Weakness = 2 points; Duration: 10–59 minutes = 1 point, >60 minutes = 2 points; Diabetes = 1 point. The blood pressure item was excluded since this was not likely to be available by self-report.

### Reported Symptoms

Speech, motor, and sensory symptoms were commonly reported (Table 1). Double vision and associated headache were also commonly reported as well. A total of 102 (58%) participants reported that symptoms were sudden in onset (onset over seconds to minutes) and 31 (19%) reported having a warning or prodrome of impending symptoms. Some participants (71; 41%) reported symptoms that had lasted for more than an hour.

A majority of participants reported that the symptoms were new within the past year (104; 59%) but 26 (15%) reported that they had had more than 11 episodes in the past year. Most participants (131; 77%) had experienced symptoms within a month and 37 (22%) had experienced symptoms within 24 hours. A substantial number of participants (71; 41%) had not yet sought formal medical advice.

### Outcomes

Based on the follow-up telephone calls, 43 (25%) participants had a probable or definite TIA, and 68 (39%) participants had a probable or definite cerebrovascular event (either stroke or TIA). There was 96% agreement between the two vascular neurologists for the diagnosis of TIA/stroke (quadratic-weighted kappa = 0.86; see Table S1) among the 84 (48%) participants that had a second independent assessment.

### Assessment of ABCD^2^ Score Components

Univariable associations between individual components of the ABCD^2^ score and TIA/stroke are presented in Table 2. A longer duration of symptoms was significantly associated with the ultimate diagnosis of TIA/stroke (Table 2). Unilateral weakness was also associated with a nearly 2-fold higher odds of TIA/stroke (OR 1.95, 95% CI 1.08−3.68).

**Table 2:** Univariable Predictors of Cerebrovascular Outcomes Among 175 Users Searching for Information on Transient Ischemic Attack and Mini-Stroke Online.

		TIA/Stroke	
		OR (95% CI)	*P*
Age > = 60		1.15 (0.63,2.13)	0.65
Clinical features			
Speech disturbance		1.35 (0.73,2.50)	0.34
Unilateral weakness		1.99 (1.06,3.76)	0.03
Duration of symptoms			
less than 10 minutes		ref	
10–59 minutes		3.21 (1.35,7.61)	<0.01
60 or more minutes		3.22 (1.46,7.11)	<0.01
Diabetes		1.63 (0.76,3.51)	0.21
Male		1.23 (0.65,2.30)	0.52
Hypertension		1.00 (0.54,1.85)	0.99
Prior TIA		1.84 (0.93,3.65)	0.08
Prior stroke		0.85 (0.38,1.93)	0.70
History of migraine		1.00 (0.53,1.88)	0.99
Timing of enrollment			
within 24 hours of symptoms		ref	
within 48 hours of symptoms		1.51 (0.41,5.56)	0.54
within 1 week of symptoms		2.86 (1.01,8.06)	0.05
within 1 month of symptoms		2.37 (0.88,6.39)	0.09
within 6 months of symptoms		4.23 (1.55,11.5)	<0.01
Number of episodes within the past year			
1		ref	
2		0.26 (0.10,0.67)	<0.01
3 to 5		0.23 (0.08,0.65)	<0.01
6 to 10		0.26 (0.09,0.83)	0.02
11 or more		0.09 (0.02, 0.33)	<0.01
At least one previous episode within past year		0.20 (0.11, 0.39)	<0.01
Sudden onset		0.77 (0.42,1.43)	0.41
Warning before episode		0.89 (0.40,1.95)	0.77
Associated headache		0.85 (0.46,1.58)	0.62
Double vision		1.65 (0.84,3.27)	0.15
Sensory symptoms		1.63 (0.88,3.01)	0.12
Already sought medical attention		2.77 (1.38, 5.59)	5.59

**Figure 1:**
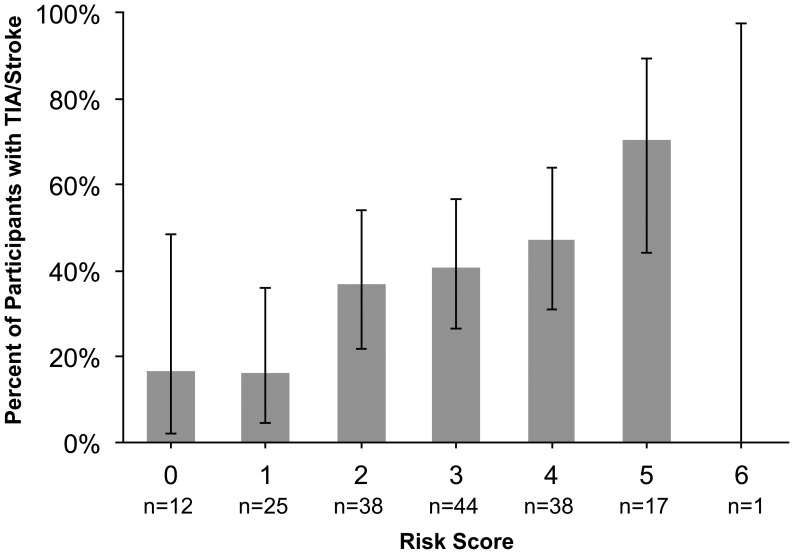
Percent of participants with transient ischemic attack or stroke by self-reported ABCD^2^ score. A modified ABCD^2^ score was calculated from self-reported components of the score (age > = 60 = 1 point; Clinical Features: Speech Disturbance without weakness = 1 point, Unilateral Weakness = 2 points; Duration: 10–59 minutes = 1 point, >60 minutes = 2 points; Diabetes = 1 point). The blood pressure item was excluded since this was not likely to be available by self-report, so the range of this risk score was 0 to 6. The grey bars show the proportion observed and the black vertical lines represent the 95% confidence interval around that proportion. The one enrollee with a score of 6 had migraine.

The mean modified ABCD^2^ score (excluding blood pressure) was 2.7, (SD 1.4; range 0–6). The proportion of patients with a TIA or stroke stratified by risk score is shown in Figure 1. Of the 12 patients with a score of 0, two (17%) had a TIA/stroke–one participant had diplopia and unilateral facial numbness that was concerning for posterior circulation ischemia, and the other had amaurosis fugax.

### Other Predictors

Participants with multiple episodes of similar symptoms within the past year were much less likely to be diagnosed with TIA/stroke (Table 2). The odds of a TIA/stroke diagnosis were nearly five times higher (OR 4.90, 95% CI 2.56−9.09) for participants with new symptoms within the last year as compared to those who had at least one previous episode of similar symptoms in the last year.

There was moderate agreement (Cronbach’s alpha 0.79) among the three items on the sudden symptom onset. However, sudden symptom onset was not significantly associated with a TIA/stroke diagnosis. Those who had already sought medical advice for their symptoms were significantly more likely to have TIA/stroke (OR 2.77, 95% CI 1.38–5.59).

### Multivariable Models

For Model 1, we included components of the ABCD^2^ score except of blood pressure (Table 3). This self-reported risk score demonstrated poor discrimination for TIA/stroke (0.66, 95% CI 0.58–0.74).

**Table 3:** Multivariable Predictors of TIA/Stroke Diagnosis Among 175 Users Searching for Information on Transient Ischemic Attack or Mini-Stroke Symptoms Online.

		TIA/Stroke	
		OR (95% CI)	*P*
Model 1: Components of the ABCD^2^ score excluding blood pressure			
Age > = 60		1.47 (0.76, 2.85)	0.25
Clinical features			
Speech Disturbance		1.09 (0.48, 2.49)	0.84
Unilateral weakness		1.79 (0.80, 3.96)	0.15
Duration of symptoms			
10–59 minutes		2.78 (1.13, 6.81)	0.03
>60 minutes		3.02 (1.32, 6.92)	<0.01
Diabetes		1.53 (0.69, 3.39)	0.30
Model 2: Model 1+ no previous episodes			
Age > = 60		1.25 (0.61, 2.56)	0.54
Clinical features			
Speech Disturbance		1.10 (0.42, 2.46)	0.98
Unilateral weakness		1.93 (0.81, 4.56)	0.14
Duration of symptoms			
10–59 minutes		3.05 (1.15, 8.07)	0.03
>60 minutes		2.39 (0.98, 5.82)	0.06
Diabetes		2.58 (1.05, 6.37)	0.04
No previous episodes (within 1 year)		5.97 (2.80, 12.7)	<0.01

Components of the ABCD^2^ score excluding acute blood pressure are included in Model 1. Model 2 adds the number of previous episodes of similar symptoms in the past year.

For Model 2, we incorporated a categorical variable with the number of episodes of similar symptoms within the past year (Table 3). This model produced a higher c-statistic (0.76, 95% CI 0.68–0.83) for TIA/stroke. However the same two participants with a risk score of 0 described above would still have been classified in the lowest risk category.

## Discussion

Using an efficient and largely automated approach, we were able to readily identify a selected group of individuals who were seeking information on recent symptoms of TIA/stroke online–sometimes very soon after symptom onset and often before a formal medical evaluation had been completed. Although participants in our study are unlikely to be fully representative of patients with TIA generally, or patients seeking online information on TIA specifically, we did identify a subpopulation with a relatively high burden of cerebrovascular disease that could benefit from a targeted public health intervention. Given the low cost and the substantial efficiency of this approach in terms of cost per participant reached, and since a major goal of acute management of TIA is urgent evaluation to prevent early recurrent stroke, this subpopulation may present an attractive target for interventions designed to reduce delays in presenting to medical attention for TIA/stroke.

A secondary goal of our study was to assess the performance of a self-reported version of ABCD^2^ score for TIA. The ABCD^2^ score was originally developed to assess the risk of stroke after TIA using simple elements available to front-line clinicians, [3] but this score has also been shown to work in part by distinguishing TIA from TIA mimics. [18] Here, we faced the additional challenge from the measurement issues that arise from using self-reported responses from individuals with various levels of health literacy. Accordingly, we found that a modified ABCD^2^ score by self-report could not reliably rule out a TIA or stroke. Some participants (2 of 12) with the lowest score of 0 had symptoms such as diplopia or amaurosis fugax that are not specifically captured by the score, but would still require urgent evaluation.

With regards to the performance of other self-reported items to predict a cerebrovascular diagnosis, we found a strong association between a higher number of episodes of similar symptoms within the last 12 months and a non-cerebrovascular diagnosis. Having multiple stereotyped episodes of symptoms previously without a resultant stroke may make a given episode less likely to be TIA and may make seizure, migraine or other episodic disorders more likely. [19] The predictive value of this finding will require further validation and confirmation of the dose-response relationship that is suggested by our data.

Since such a high proportion of participants in our study had TIA or stroke, we speculate that all of the individuals that participated in our study may have been well served to seek medical attention regardless of risk score, particularly since the online questionnaire was unable to reliably identify low-risk individuals. Some individual participants indicated that they lacked a primary care provider or medical insurance or otherwise had limited access to medical care, while others indicated that they did not understand that TIA was a medical emergency, or were unable to get an urgent appointment, but had already planned on seeking medical attention at some point. The value of some form of risk stratification at the outset may not be to identify those without worrisome symptoms, but to provide some interactivity and engagement in order to enhance behavior change while participants are deciding on next actions. We suspect that if individuals are given feedback and recommendations that are to some extent individualized and interactive rather than presented as static blanket recommendations, they may be more likely to take action after TIA, though a formal assessment of this possibility would require additional study.

Our methods also highlight opportunities to improve the efficiency of clinical research more generally by quickly and efficiently reaching hundreds of potential participants on a daily basis and by enrolling participants over a large geographic area. Direct-to-participant recruitment portals that incorporate initial eligibility screening by self-report may demonstrate a sufficiently high yield for TIA and stroke diagnoses to serve as a primary recruitment method for some types of clinical research. Furthermore, our study frames the potential role for validated and interactive self-assessment online tools address public health problems more generally. A previous study have demonstrated a relationship between internet search queries and influenza epidemics [20] and another study showed that the frequency of internet search queries for stroke related search terms by state (“stroke signs”, “stroke symptoms”, “mini stroke”, excluding “heat”) was associated with stroke prevalence. [21] There remain potential medicolegal issues including whether providing tailored or personalized information constitutes medical advice, and valid concerns about maintaining privacy and confidentiality of health information online. But as disparities in access [22], [23], [24] and adoption of internet technology across demographic and sociodemographic strata narrow, [14] internet-based interventions to efficiently target public health problems may become more attractive. Future studies may include questionnaires in the ED regarding internet-based activity prior to presentation in the ED and evaluations of the impact of internet-based tools on the actual the behavior of subjects with possible TIA and ultimately on processes measures of treatment and patient outcomes.

### Limitations

Our study should be interpreted in light of a number of limitations. First, about 1% of visitors to the website enrolled in the study and so the participants in this study were highly selected. Although the motivations for enrollment were not captured in our study, many visitors quickly moved from the website within a few seconds, which suggests that they were not finding what they had expected or what they were looking for. We speculate that individuals with symptoms without a straightforward diagnosis despite prior medical evaluations, those with chronic medical conditions, or those with barriers to accessing care might be overrepresented in our study. Second, the authentication of participants and their responses and the difficulty of reaching some participants by telephone limit the generalizability of our results and illustrate a particular challenge for internet-based research. Third, we attempted to mitigate misclassification of TIA and stroke outcomes by formally assessing interrater reliability and masking the neurologists to questionnaire responses, but since follow-up was conducted by telephone and access to additional clinical information such as neuroimaging studies or results of a neurologic exam was limited to self-report the potential for misclassification remains. For the lay-public, the use of the term, “mini-stroke,” a much more common search term than “TIA”, serves to confuse the distinction between TIA and stroke and the clinical distinction between TIA and minor stroke may be difficult to make even for experienced clinicians. [25], [26] Fourth, although the neurologists were masked to responses to the questionnaire, they were free to elicit any information into their final assessments and may have incorporated items that were elicited by the online questionnaire into their assessments.

### Conclusion

Individuals seeking information on possible TIA or stroke symptoms online can be efficiently and readily identified, sometimes before they have sought formal medical attention. Although elements of a self-reported ABCD^2^ score was not able to rule out a cerebrovascular etiology, the burden of true stroke and TIA in this selected subpopulation may be sufficiently high to justify future targeted interventions to encourage urgent medical evaluation.

## Supporting Information

Table S1 **Interrater agreement on cerebrovascular diagnosis.** Agreement between two independent telephone assessments by vascular neurologists for the diagnosis of stroke/TIA among 84 participant seeking information on TIA/stroke symptoms on the internet. Agreement for Stroke/TIA was 95.8% and the quadratic-weighted kappa was 0.86.
(PDF)

Text S1 **Internet Advertisements.**
(PDF)

Text S2 **Target Search Terms for Targeting Internet Advertisements.**
(PDF)

Text S3 **Questionnaire.**
(PDF)
