# Supplementary material for: A Cross-Sectional Study of Individuals Seeking Information on Transient Ischemic Attack and Stroke Symptoms Online: A Target for Intervention?
Source: PLoS One. 2012 Oct 31;7(10):e47997. doi: 10.1371/journal.pone.0047997 (PMC3485263; doi:10.1371/journal.pone.0047997)
Supplement: Table S1 — Interrater agreement on cerebrovascular diagnosis. Agreement between two independent telephone assessments by vascular neurologists for the diagnosis of stroke/TIA among 84 participant seeking information on TIA/stroke symptoms on the internet. Agreement for Stroke/TIA was 95.8% and the quadratic-weighted kappa was 0.86. (PDF) [file pone.0047997.s001.pdf]

Neurologist 2 \ Neurologist 1	Definitely not a Stroke/TIA	Unlikely to be a Stroke/TIA	Probable Stroke/TIA	Definite Stroke/TIA	Total
**Definitely not a Stroke/TIA**	21	13	1	0	**35**
**Unlikely to be a Stroke/TIA**	3	11	2	0	**16**
**Probable Stroke/TIA**	0	1	9	1	**11**
**Definite Stroke/TIA**	0	0	8	14	**22**
**Total**	**24**	**25**	**20**	**15**	**84**
