# Supplementary material for: A Cross-Sectional Study of Individuals Seeking Information on Transient Ischemic Attack and Stroke Symptoms Online: A Target for Intervention?
Source: PLoS One. 2012 Oct 31;7(10):e47997. doi: 10.1371/journal.pone.0047997 (PMC3485263; doi:10.1371/journal.pone.0047997)
Supplement: Text S1 — Internet Advertisements. (PDF) [file pone.0047997.s002.pdf]

Text S1 - Internet Advertisements

Mini-Stroke Symptom Study
Learn about whether your symptoms
could be from a mini-stroke or TIA.
tia.ucsf.edu

Possible Mini-Stroke/TIA?
Online research study to evaluate
symptoms of TIA or mini-stroke
tia.ucsf.edu
