# Supplementary material for: A Cross-Sectional Study of Individuals Seeking Information on Transient Ischemic Attack and Stroke Symptoms Online: A Target for Intervention?
Source: PLoS One. 2012 Oct 31;7(10):e47997. doi: 10.1371/journal.pone.0047997 (PMC3485263; doi:10.1371/journal.pone.0047997)
Supplement: Text S2 — Target Search Terms for Targeting Internet Advertisements. (PDF) [file pone.0047997.s003.pdf]

Text S2 - Target Search Terms for Targeting Internet Advertisements

tias mini strokes
tia mini stroke symptoms
effects of mini strokes
trans ischemic attacks
symptoms of mini strokes
symptoms of a mini stroke
transient ischemic attack mini stroke
mini stroke symptoms
what causes a mini stroke
signs of mini stroke
signs of mini strokes
signs of a minor stroke
mini stroke symptom
mini stroke signs
signs of a mini stroke
transient ischemic attack stroke
mini stroke causes
symptoms of small stroke
symptoms of a tia stroke
mini strokes symptoms
mini stroke
symptoms of transient ischemic attack
mini stroke signs and symptoms
symptom of a stroke
signs and symptoms of stroke in women
what are symptoms of a stroke
symptoms of mini stroke
the symptoms of a stroke
signs of strokes
tia strokes causes
symptoms of mild stroke
trans ischemic attack
symptoms of having a stroke
symptoms of tia stroke
signs before a stroke
tia stroke
symptoms of tia
early stroke symptoms
transient ischemic
ischemic attack symptoms
stroke symptoms in women
signs of a mild stroke
symptom of stroke
signs of an impending stroke
5 signs of a stroke
stroke symptoms warning signs
transient attack
mini stroke treatment
tia stroke symptoms
what causes mini strokes
sign of a stroke
ischemic attack
tias symptoms
mild stroke symptoms
signs of a stroke
symptoms stroke
causes of mini strokes
what is a tia
mini stroke in women
what are the symptoms of stroke
signs of impending stroke
what are symptoms of stroke
symptoms for a stroke
sign of strokes
early signs of a stroke
sign of stroke
transient ischemic attacks symptoms
signs of a tia stroke
what are stroke symptoms
small strokes symptoms
mini strokes in women
warning signs of stroke
small stroke symptoms
mini strokes
symptoms for stroke
warning signs of a stroke
mini stroke symptoms in men
signs of mild stroke
common stroke symptoms
symptoms before a stroke
mini stroke stroke
sign of stroke in women
mini strokes in elderly
signs of mini stroke in women
causes of a stroke
pre stroke symptoms
what is transient ischemic attack
symptoms of a mild stroke
signs for stroke
transient ischemic attack symptoms
symptoms of stroke in men
stroke like symptoms
symptoms of stroke in women
having a stroke
early symptoms of a stroke
transient ischemic attacks
signs stroke
symptoms of a minor stroke
the symptoms of stroke
symptoms of mini stroke in women
signs symptoms of a stroke
signs of stroke in women
early warning signs of stroke
signs and symptoms of a stroke
signs of stroke
strokes symptoms
symptoms of a stroke in women
am i having a stroke
early symptoms of stroke
symptoms of a stroke

symptoms of strokes
3 signs of a stroke
what are signs of stroke
stroke warning signs
women stroke symptoms
what is a transient ischemic attack
what are the symptoms of a stroke
symptoms of a mini stroke in women
what is a stroke
early signs of stroke
signs of having a stroke
signs symptoms stroke
mini stroke symptoms for women
stroke signs and symptoms
symptoms of stroke
signs of a stroke in a woman
pre stroke signs
cva symptoms
transient ischemic attack tia
sign and symptoms of stroke
signs and symptoms of stroke
ischemic stroke symptoms
symptoms before stroke
warning signs of stroke in women
symptoms of strokes in women
mini stroke diagnosis
signs of a small stroke
4 signs of a stroke
signs your having a stroke
symptoms of ischemic stroke
cause of a stroke
mild stroke symptoms for women
signs and symptoms of a stroke in women
stroke symptoms and treatment
symptom of stroke in women
symptoms of a small stroke
transient ischemic stroke symptoms
what are the signs and symptoms of a stroke
five signs of stroke
5 warning signs of stroke
causes of stroke in women
