# Supplementary material for: A Cross-Sectional Study of Individuals Seeking Information on Transient Ischemic Attack and Stroke Symptoms Online: A Target for Intervention?
Source: PLoS One. 2012 Oct 31;7(10):e47997. doi: 10.1371/journal.pone.0047997 (PMC3485263; doi:10.1371/journal.pone.0047997)
Supplement: Text S3 — Questionnaire. (PDF) [file pone.0047997.s004.pdf]

Text S3 - Questionnaire

PLEASE ANSWER THESE QUESTIONS AS BEST AS YOU CAN.

1. How old are you? (in years)

2. What is your gender?
- Male
- Female

3. The following questions are about medical problems that you were diagnosed with in the past, before you developed TIA or mini-stroke symptoms.

3a. Have you ***ever*** been told by a doctor or nurse that you had diabetes (sugar diabetes)?
- Yes
- No

3b. Are you ***currently*** taking medicine for diabetes (sugar diabetes)?
- Yes
- No

3c. Have you ***ever*** been told by a doctor or nurse that you had high blood pressure (hypertension)?
- Yes
- No

3d. Are you ***currently*** taking medicine for high blood pressure (hypertension)?
- Yes
- No

3e. Have you ever been told by a doctor or nurse that you had a stroke in the past (not including this most recent episode)?
- Yes
- No

3f. Have you ***ever*** been told by a doctor or nurse that you had a mini-stroke, TIA, or transient ischemic attack in the past (**not** including this most recent episode)?
- Yes
- No

3g. Has a doctor or nurse ***ever*** told you that you have migraines?
- Yes
- No

4. The following questions are about the specific episode of symptoms that are concerning for mini-stroke or TIA episode. If you have had multiple episodes of symptoms in the past, choose the most recent episode of symptoms that is the most concerning to you.

4a. How long ago did these symptoms occur?

- ◯ Within the last 24 hours
- ◯ Within the last 48 hours
- ◯ Within the last week
- ◯ Within the last month
- ◯ Within the last six months

4b. How long did your symptoms last?

- ◯ Less than 10 minutes
- ◯ 10-59 minutes
- ◯ 60 minutes or more

4c. How many times has this same set of symptoms occurred in the last 12 months?

- ◯ only once
- ◯ twice
- ◯ 3 to 5 times
- ◯ 6 to 10 times
- ◯ 11 or more times

4d. Were your symptoms at their worst within seconds of onset?

- ◯ Yes
- ◯ No

4e. Did your symptoms gradually worsen over minutes to hours?

- ◯ Yes
- ◯ No

4f. How did your symptoms start?

- ◯ Yes
- ◯ No

4g. Did you ***suddenly*** have weakness on only one side of your body?

- ◯ Yes
- ◯ No

4h. Did you have trouble moving your arms or legs?

- ◯ Yes
- ◯ No

4i. Did you have any warning that an episode was about to occur?

- ◯ Yes
- ◯ No

4j. Did you ***suddenly*** lose the ability to express yourself verbally or in writing?

◯ Yes
◯ No

4k. Did you ***suddenly*** lose the ability to understand what people were saying?

◯ Yes
◯ No

4l. Did you have slurred speech during the episode?

◯ Yes
◯ No

4m. Did you have trouble speaking during the episode?

◯ Yes
◯ No

4n. Did you have ***sudden*** numbness on only one side of your body?

◯ Yes
◯ No

4o. Did you develop a headache right before, during, or right after the episode?

◯ Yes
◯ No

4p. Did you have any of the following during the episode (Check all that apply)

☐ Sudden weakness
☐ Sudden numbness
☐ Sudden double vision
☐ Sudden trouble speaking
☐ Sudden trouble understanding
☐ None of the above.

4q. Have you already sought medical advice about this episode?

◯ Yes
◯ No
